# Stair climbing and the incidence of atherosclerotic cardiovascular disease: a population-based prospective cohort study

**DOI:** 10.1265/ehpm.23-00166

**Published:** 2023-10-27

**Authors:** Ahmed Arafa, Rena Kashima, Yoshihiro Kokubo

**Affiliations:** 1 Department of Preventive Cardiology, National Cerebral and Cardiovascular Center, Suita, Japan; 2 Department of Public Health, Faculty of Medicine, Beni-Suef University, Beni-Suef, Egypt; 3 Department of Cardiovascular Pathophysiology and Therapeutics, Graduate School of Medicine, Osaka University, Suita, Japan

**Keywords:** Stair climbing, ASCVD, Prospective cohort, Population-based study

## Abstract

**Background:**

Stair climbing is a readily available form of physical activity with potential cardioprotective merits. Herein, we investigated the association between stair climbing and atherosclerotic cardiovascular disease (ASCVD) incidence among Japanese people.

**Methods:**

This prospective cohort study used data from 7,282 participants, aged 30–84 years, registered in the Suita Study and free from stroke and ischemic heart disease (IHD). Standard approaches were used to detect incident ASCVD events, including cerebral infarction and IHD, during follow-up. Stair climbing was assessed using a baseline questionnaire. We applied the Cox regression to calculate the hazard ratios (HRs) and 95% confidence intervals (95% CIs) of incident ASCVD for climbing stairs in 20–39%, 40–59%, and ≥60% compared to <20% of the time. We adjusted the regression models for age, sex, body mass index, smoking, alcohol consumption, physical activity, hypertension, diabetes, atrial fibrillation, lipid profile, chronic kidney disease, and history of cardiac murmur or valvular diseases.

**Results:**

A total of 536 new ASCVD events were detected within a median follow-up period of 16.6 years. In the age- and sex-adjusted model, stair climbing 20–39%, 40–59%, and ≥60% of the time was associated with lower ASCVD incidence: HRs (95% CIs) = 0.72 (0.56, 0.92), 0.86 (0.68, 1.08), and 0.78 (0.61, 0.99), respectively (p-trend = 0.020). The corresponding associations were attenuated after adjusting for lifestyle and clinical factors: HRs (95% CIs) = 0.74 (0.58, 0.95), 0.90 (0.71, 1.13), and 0.89 (0.69, 1.13), respectively (p-trend = 0.152).

**Conclusion:**

Frequent stair climbing was associated with lower ASCVD incidence; however, this association was partly explained by lifestyle and clinical factors of participants.

**Supplementary information:**

The online version contains supplementary material available at https://doi.org/10.1265/ehpm.23-00166.

## 1. Introduction

Atherosclerotic cardiovascular disease (ASCVD) remains the leading cause of morbidity and mortality in Japan and globally [1]. However, identifying modifiable risk factors for ASCVD may help reduce its burden [2]. Physical activity (PA) is a modifiable lifestyle behavior with documented cardioprotective benefits [2]. Among different PA forms, stair climbing is a good means of accumulating PA that many people perform regularly [3]. Previous studies showed that stair climbing could reduce the risk of obesity, diabetes, hypertension, metabolic syndrome, and atrial fibrillation; factors closely related to ASCVD [4–8]. It can also improve endothelial and vascular smooth muscle functions in patients with hypertension [9], cardiorespiratory fitness in patients with ischemic heart disease (IHD) [10], and cognitive performance in healthy young adults [11].

A few studies investigated the association between stair climbing and CVD [12–15]. However, in addition to their scarcity and inconsistent findings, these studies investigated CVD mortality rather than incidence. Causes of death are often determined based on death certificates, which may not always accurately reflect the underlying cause, leading to biased risk estimates. In addition, the risk of CVD mortality changes over time due to improvements in medical treatments. Ford et al. estimated that 47% of the reduction in IHD mortality rate between 1980 and 2000 was explained by medical and surgical treatments, while the control of CVD risk factors contributed to 44% of this reduction [16]. Thus, baseline lifestyle data collected in epidemiological studies in earlier periods may not accurately predict CVD mortality.

Of note, we previously investigated the association between stair climbing and the risk of all-cause, cancer, and CVD mortality among older adults (≥60 years) from the Suita Study [15]. However, extrapolating the findings of the previous study to the whole population was challenging for three main reasons. First, the analysis was limited to older adults. Second, CVD mortality risk could have been significantly affected by the advancement in CVD treatment. Third, the limited number of participants by stair climbing frequencies might have weakened the statistical power.

In this context, we used data from the Suita Study to investigate the association between stair climbing and ASCVD incidence among Japanese adults ≥30 years.

## 2. Methods

### 2.1. Study design and participants

We reported this study per the guidelines of the Strengthening the Reporting of Observational studies in Epidemiology (STROBE) [17]. As described elsewhere [18], the Suita Study is a prospective cohort study aiming to investigate CVD risk factors among urban Japanese people. A total of 8,360 people from the Japanese city of Suita were recruited. The Suita population included two randomly selected cohorts by 10-year age and sex (recruited between 1989 and 1998) and a volunteer group (recruited between 1992 and 2006). All participants underwent baseline health examination which included collecting data about personal, lifestyle, and clinical characteristics, blood sampling, clinical assessment, and undertaking ECG. Then, participants were asked to return every couple of years for follow-up. In this study, we excluded 1,078 participants for either having a positive history of stroke or IHD, lacking baseline data about stair climbing, or were lost to follow-up, leaving 7,282 participants (3,376 men and 3,906 women), aged 30–84 years, for analysis.

### 2.2. Outcome, exposure, and covariates

ASCVD consisted of incident cerebral infarction and IHD, including myocardial infarctions, percutaneous coronary intervention, coronary artery bypass grafting, and sudden cardiac death. Cerebral infarctions were diagnosed per the criteria of the US National Survey of Stroke according to CT and MRI scans [19], while myocardial infarctions were diagnosed per the criteria of the World Health Organization MONICA Project (monitoring trends and determinants in cardiovascular disease) [20].

As described elsewhere [8], stair climbing was assessed using a question in the Suita Study baseline questionnaire: *“In public and private buildings with stairs and escalators or elevators, what do you usually use to climb up to the height of the third floor or higher?”* The responses were *“I use stairs most of the time (Stairs ≥ 80%)”, “I use stairs more than escalators or elevators (Stairs 60–79%)”, “half-half (Stairs 40–59%)”, “I use escalators or elevators more than stairs (Stairs 20–39%)”,* and *“I use escalators or elevators most of the time (Stairs < 20%)”*.

Covariates were assessed during the baseline medical check-up. Hypertension was defined as blood pressure >140/90 mmHg or receiving medications. Diabetes was defined as fasting blood glucose ≥126 mg/dL or receiving medication. Chronic kidney disease was defined as an estimated glomerular filtration rate <60 mL/min/1.73 m^2^. Atrial fibrillation was determined based on ECG and medical records. PA was assessed using a simple question “*Do you practice physical activity for your health?*”, and the possible responses were “*yes*” and “*no*”.

### 2.3. Statistical analysis

We merged the responses of 60–79% and ≥80% into one group of ≥60% due to the relatively limited number of participants in both groups. The Chi-squared test was applied to assess the differences in the baseline characteristics among different stair-climbing groups. The Cox proportional hazards models were used to calculate the hazard ratios (HRs) and their 95% confidence intervals (95% CIs) of ASCVD for participants who reported stair climbing 20–39%, 40–59%, and ≥60% of the time compared to those who reported stair climbing <20% of the time. Further, we stratified the results by age, sex, body mass index (BMI), and PA. Person-years were calculated from the date of baseline assessment until the date of ASCVD event, death, leaving the study, or the end of follow-up (December 31, 2013), whichever came first. We adjusted the regression models for the following variables: age (<50, 50–59, 60–69, or ≥70 years), sex, BMI (<18.5, 18.5–24.9, ≥25 kg/m^2^), smoking (non-current or current), alcohol consumption (non-current or current), PA (yes or no), hypertension (yes or no), diabetes (yes or no), atrial fibrillation (yes or no), total cholesterol (TC) (<200, 200–239, or ≥240 mg/dL), high-density lipoprotein-cholesterol (HDL-C) (<40, 40–59, or ≥60 mg/dL), chronic kidney disease (CKD) (yes or no), and cardiac murmur or valvular diseases (yes or no). SAS version 9.4 software (SAS Institute Inc, Cary, NC) was used for statistical analyses.

## 3. Results

Participants who reported higher frequencies of stair climbing were younger and less likely to have obesity, hypertension, and CKD, but more likely to be smokers (p-values < 0.05) (Table 1). Within 108,351 person-years (median follow-up = 16.6 years), 536 incident ASCVD events were detected. In the age- and sex-adjusted model, stair climbing 20–39%, 40–59%, and ≥60% of the time was associated with lower ASCVD incidence: HRs (95% CIs) = 0.72 (0.56, 0.92), 0.86 (0.68, 1.08), and 0.78 (0.61, 0.99), respectively (p-trend = 0.020). The corresponding associations were attenuated in the multivariable-adjusted model: HRs (95% CIs) = 0.74 (0.58, 0.95), 0.90 (0.71, 1.13), and 0.89 (0.69, 1.13), respectively (p-trend = 0.152) (Table 2). Stratifying the results by age, sex, BMI, and PA did not significantly affect the results (p-interactions > 0.10) (Supplementary Table 1).

**Table 1:** Characteristics of participants by stair climbing

**Characteristics**		**Stair climbing**				

		**<20%**	**20–39%**	**40–59%**	**≥60%**	**P-difference**
Number of participants		1,773	1,787	1,967	1,755	—
Sex, %	Men	41.0	42.4	45.0	57.3	<0.001
	Women	59.0	57.6	55.0	42.7	
Age, %	<50 years	33.2	36.4	33.3	34.9	<0.001
	50–59 years	20.3	21.5	25.4	26.8	
	60–69 years	21.8	24.8	27.1	25.9	
	≥70 years	24.7	17.3	14.2	12.4	
Body mass index, %	<18.5 kg/m^2^	8.6	8.6	7.5	8.4	<0.001
	18.5–24.9 kg/m^2^	67.6	71.1	73.5	74.9	
	≥25 kg/m^2^	23.8	20.3	19.0	16.7	
Current smokers, %		29.7	26.5	26.8	33.1	<0.001
Current drinkers, %		53.2	50.8	48.4	42.1	<0.001
Physical activity, %		31.3	36.8	43.2	50.0	<0.001
Hypertension, %		34.4	30.7	33.6	28.9	0.001
Diabetes, %		6.3	5.2	5.0	5.0	0.243
High-density lipoprotein-cholesterol, %	<40 mg/dL	15.6	14.2	13.9	14.2	0.525
	40–59 mg/dL	52.3	50.8	52.6	52.0	
	≥60 mg/dL	32.1	35.0	33.5	33.8	
Total cholesterol, %	<200 mg/dL	42.3	42.3	40.5	44.8	0.189
	200–239 mg/dL	38.9	39.4	40.1	38.5	
	≥240 mg/dL	18.8	18.3	19.4	16.7	
Chronic kidney disease, %		9.7	8.9	7.6	7.0	0.014
Cardiac murmur, %		2.7	2.7	2.5	2.1	0.533
Atrial fibrillation, %		1.8	1.6	1.1	1.0	0.142

**Table 2:** Stair climbing and the risk of atherosclerotic cardiovascular disease

**Stair climbing**	**Incident cases**	**Incidence/1,000 ** **person-years**	**Model I** **HR (95% CI)**	**Model II** **HR (95% CI)**
<20%	148	5.9	1 (Ref)	1 (Ref)
20–39%	109	4.1	0.72 (0.56, 0.92)	0.74 (0.58, 0.95)
40–59%	151	5.1	0.86 (0.68, 1.08)	0.90 (0.71, 1.13)
≥60%	128	4.8	0.78 (0.61, 0.99)	0.89 (0.69, 1.13)
P-trend	—	—	0.020	0.152

Model I: adjusted for age and sex

Model II: adjusted further for BMI, smoking, alcohol consumption, physical activity, hypertension, diabetes, AF, HDL-C, TC, CKD, and cardiac murmur

HRs (95% CIs) for stair climbing ≥20% compared to <20% were as follows: Model I: 0.79 (0.65, 0.95) and Model II: 0.84 (0.69, 1.02)

## 4. Discussion

This study indicated that frequent stair climbing could be associated with a decreased risk of incident ASCVD among Japanese people. However, the attenuation of the cardioprotective effect of stair climbing after adjustment for lifestyle and clinical factors and the absence of a dose-response relationship across stair-climbing frequencies may suggest that this association is partly explained by covariates.

A few studies investigated the association between stair climbing and CVD mortality, not incidence, and reached contradictory conclusions. The Harvard Alumni Health Study showed that stair climbing of 10–19, 20–34, and ≥35 floors/week compared to <10 floors/weeks was not associated with CVD mortality: HRs (95% CIs) = 0.94 (0.79, 1.11), 0.90 (0.76, 1.08), and 0.94 (0.81, 1.09), respectively [12]. Alike, the UK Biobank study showed that stair climbing of 1–5, 6–10, 11–15, and ≥16 flights (around 10 stairs)/day compared to no stair climbing was not associated with CVD mortality: HRs (95% CIs) = 1.14 (0.95, 1.37), 1.08 (0.91, 1.29), 1.05 (0.86, 1.28), and 1.04 (0.84, 1.28), respectively [13]. In a recent report from the Suita Study, we detected an inverse association between stair climbing 40–59% of the time and CVD mortality in older adults, but the associations were not statistically significant in lower or higher stair climbing frequencies: HRs (95% CIs) = 0.80 (0.59, 1.07), 0.59 (0.43, 0.80), 0.86 (0.58, 1.27), and 0.82 (0.56, 1.20) for the frequencies 20–39%, 40–59%, 60–79%, and ≥80% of the time [15]. However, the three studies showed statistically-significant inverse associations between stair climbing and all-cause mortality [12, 13, 15]. On the other hand, among patients with peripheral arterial disease in Chicago, those in the lowest quartile of a score for stair climbing showed higher CVD mortality compared to those in the highest quartile: HR (95% CI) = 1.70 (1.08, 2.66) [14].

Of note, stair-climbing promotion interventions could be effective. A previous study showed that using signs and enhancement of stairwell aesthetics increased workplace stair climbing by 18.7% in a corporate building in Paris [21]. Another intervention program comprising health awareness sessions was able to increase daily stair climbing among Japanese older men and women by 47 and 36 steps/day, respectively [22]. Further, improving the visibility of the stairwell and displaying a stair-climbing video on a screen between the stairs and the elevator increased stair climbing in a Belgian worksite by 6% and 12.5%, respectively [23].

This study had several strengths, such as including a representative sample of urban Japanese people, using a prospective cohort design to allow for a temporal association to be investigated, having a long follow-up period to minimize the risk of reverse causality, applying standardized methods for ASCVD diagnosis, and controlling the results for most confounders. Still, many limitations should be considered. First, we assessed stair climbing during baseline. Some participants might have changed their stair-climbing behavior during follow-up. Second, since stair climbing practice was self-reported, recall bias is possible. Third, participants were asked whether they were climbing stairs to the third floor or higher; therefore, participants who were climbing stairs to the first or second floors only might have been assigned to the <20% group. This categorization might have resulted in differential misclassification and attenuation of the cardioprotective effect of stair climbing. Fourth, climbing stairs could have been significantly influenced by family structure. It could be speculated that those who were living with infants or older adults used elevators more than stairs. Unfortunately, we do not have enough data about family structure to adjust for. Fifth, the height of floors and number of stairs per floor may vary across buildings. Sixth, we do not have details about the diseases that are characterized by mobility restriction, such as arthritis, musculoskeletal disorders, including back pain, herniated discs, and fractures, and neurological disorders, such as multiple sclerosis and amyotrophic lateral sclerosis. Seventh, we have no data about types, strengths, and durations of occupational and non-occupational PA practiced by participants.

In conclusion, frequent stair climbing was associated with a reduced risk of ASCVD among Japanese people in the age-and sex-adjusted models. However, this association was attenuated after further adjustment for lifestyle and clinical factors. Although the inverse association between stair climbing 20–39% of the time and ASCVD incidence remained statistically significant in the multivariable-adjusted model, the absence of a dose-response relationship across different frequencies of stair climbing makes it presumptive to conclude an independent relationship between stair climbing and incident ASCVD.

## Abbreviations

ASCVD: Atherosclerotic cardiovascular disease
BMI: Body mass index
BP: Blood pressure
CI: Confidence interval
CKD: Chronic kidney disease
HDL-C: High-density lipoprotein-cholesterol
HR: Hazard ratio
IHD: Ischemic heart disease
MONICA: Multinational MONItoring of Trends and Determinants in CArdiovascular Disease
STROBE: Strengthening the Reporting of Observational studies in Epidemiology
TC: Total cholesterol

## Supplementary information

**Additional file 1:** **Supplementary Table 1:** The association between stair climbing and the risk of atherosclerotic cardiovascular disease by potential confounders.

## Declarations

### Availability of data and materials

Data could be provided upon reasonable request.
