# Supplementary material for: Stair climbing and the incidence of atherosclerotic cardiovascular disease: a population-based prospective cohort study
Source: Environ Health Prev Med. 2023 Oct 27;28:60. doi: 10.1265/ehpm.23-00166 (PMC10613554; doi:10.1265/ehpm.23-00166)
Supplement: Supplementary file 1 — Additional file 1: Supplementary Table 1: The association between stair climbing and the risk of atherosclerotic cardiovascular disease by potential confounders. [file ehpm-28-060-s001.docx]

Supplementary Table 1: The association between stair climbing and the risk of atherosclerotic cardiovascular disease by potential confounders

| Variables | Stair climbing | | | | |
| --- | --- | --- | --- | --- | --- |
|  | <20% | 20-39% | 40-59% | ≥60% | P- interaction |
| Age <65 years | 1 (Ref) | 0.82 (0.57, 1.19) | 0.96 (0.69, 1.33) | 0.98 (0.70, 1.38) | 0.735 |
| Age ≥65 years | 1 (Ref) | 0.67 (0.48, 0.95) | 0.89 (0.64, 1.22) | 0.84 (0.59, 1.19) |  |
| Men | 1 (Ref) | 0.84 (0.60, 1.17) | 1.00 (0.73, 1.36) | 0.92 (0.67, 1.26) | 0.107 |
| Women | 1 (Ref) | 0.67 (0.46, 0.98) | 0.86 (0.60, 1.23) | 0.94 (0.61, 1.43) |  |
| BMI <25 kg/m^2^ | 1 (Ref) | 0.74 (0.56, 0.99) | 0.78 (0.60, 1.03) | 0.81 (0.61, 1.07) | 0.170 |
| BMI ≥25 kg/m^2^ | 1 (Ref) | 0.71 (0.42, 1.18) | 1.31 (0.84, 2.05) | 1.21 (0.73, 1.99) |  |
| Physically active | 1 (Ref) | 0.70 (0.45, 1.09) | 0.99 (0.68, 1.44) | 0.91 (0.61, 1.34) | 0.394 |
| Physically inactive | 1 (Ref) | 0.79 (0.58, 1.07) | 0.84 (0.62, 1.13) | 0.88 (0.64, 1.22) |  |

Adjusted for age and sex, BMI, smoking, alcohol consumption, physical activity, hypertension, diabetes, AF, HDL-C, TC, CKD, and cardiac murmur
